# Gene Expression in Obliterative Bronchiolitis-Like Lesions in 2,3-Pentanedione-Exposed Rats

**DOI:** 10.1371/journal.pone.0118459

**Published:** 2015-02-24

**Authors:** Daniel L. Morgan, B. Alex Merrick, Kevin E. Gerrish, Patricia S. Stockton, Yu Wang, Julie F. Foley, William M. Gwinn, Francine L. Kelly, Scott M. Palmer, Thai-Vu T. Ton, Gordon P. Flake

**Affiliations:** 1 Division of the National Toxicology Program, National Institute of Environmental Health Sciences, Research Triangle Park, North Carolina, United States of America; 2 Division of Intramural Research, National Institute of Environmental Health Sciences, Research Triangle Park, North Carolina, United States of America; 3 Duke University Medical Center, Durham, North Carolina, United States of America; Cedars-Sinai Medical Center, UNITED STATES

## Abstract

Obliterative bronchiolitis (OB) is an irreversible lung disease characterized by progressive fibrosis in the small airways with eventual occlusion of the airway lumens. OB is most commonly associated with lung transplant rejection; however, OB has also been diagnosed in workers exposed to artificial butter flavoring (ABF) vapors. Research has been limited by the lack of an adequate animal model of OB, and as a result the mechanism(s) is unclear and there are no effective treatments for this condition. Exposure of rats to the ABF component, 2,3-pentanedione (PD) results in airway lesions that are histopathologically similar to those in human OB. We used this animal model to evaluate changes in gene expression in the distal bronchi of rats with PD-induced OB. Male Wistar Han rats were exposed to 200 ppm PD or air 6 h/d, 5 d/wk for 2-wks. Bronchial tissues were laser microdissected from serial sections of frozen lung. In exposed lungs, both fibrotic and non-fibrotic airways were collected. Following RNA extraction and microarray analysis, differential gene expression was evaluated. In non-fibrotic bronchi of exposed rats, 4683 genes were significantly altered relative to air-exposed controls with notable down-regulation of many inflammatory cytokines and chemokines. In contrast, in fibrotic bronchi, 3807 genes were significantly altered with a majority of genes being up-regulated in affected pathways. Tgf-β2 and downstream genes implicated in fibrosis were significantly up-regulated in fibrotic lesions. Genes for collagens and extracellular matrix proteins were highly up-regulated. In addition, expression of genes for peptidases and peptidase inhibitors were significantly altered, indicative of the tissue remodeling that occurs during airway fibrosis. Our data provide new insights into the molecular mechanisms of OB. This new information is of potential significance with regard to future therapeutic targets for treatment.

## Introduction

Obliterative bronchiolitis (OB) is an irreversible lung disease characterized by a progressive epithelial degeneration and obstructive fibroproliferative lesions within the small airways with eventual occlusion of the airway lumens [1]. OB is a rare disease that is most commonly associated with lung transplant rejection, although increasingly reported in the occupational setting. In particular, obstructive lung disease consistent with OB has been diagnosed in microwave popcorn packaging and flavoring industry workers exposed to artificial butter flavoring vapors containing the 4-carbon α-diketone 2,3-butanedione (diacetyl) [2]. Inhalation exposure of rats to 2,3-butanedione (BD) or to 2,3-pentanedione (PD), a 5-carbon α-diketone flavoring agent, has been shown to cause airway lesions that are histopathologically similar to OB lesions in humans [3].

The etiology of OB is unclear and there is no effective treatment, whether OB is a result of lung transplantation or inhalation of reactive chemicals. In order to develop effective therapeutic strategies, a better understanding of the molecular mechanism(s) involved in the pathogenesis of OB is needed. In this study we evaluated differential gene expression in bronchial fibrotic lesions from PD-exposed rats to help identify potential molecular pathways of airway fibrosis. Differential gene expression can be a sensitive tool for identifying pathogenic pathways; however, the resulting gene profiles can be difficult to interpret when RNA from whole tissue samples is used. Signal dilution is often a problem with lung tissue because of the large number of cell types present, and especially for lungs with OB since fibrotic airways are not uniformly distributed. In this study laser capture micro-dissection (LCM) was used to selectively harvest tissue from fibrotic and non-fibrotic bronchi from PD-exposed rats, and corresponding normal bronchial tissue from air-exposed controls. Differential gene expression in OB-like lesions of PD-exposed rats was found to be significantly up-regulated for several known profibrotic mediators, as well as for a number of genes not previously associated with OB pathogenesis.

## Materials and Methods

### Ethics statement

This study was conducted in strict accordance with the recommendations in the Guide for the Care and Use of Laboratory Animals of the National Institutes of Health. The protocol was approved by the Alion Science and Technology Animal Care and Use Committee (permit #A3016-02). All efforts were made to minimize animal suffering.

### Animals

Male Wistar-Han rats (6–7 weeks old) were obtained from Charles River Laboratories (Raleigh, NC). Animals were individually housed in polycarbonate cages for 7–10 days after arrival and were provided with food (NIH-31) and water *ad libitum*. Animals were housed in a humidity- and temperature-controlled, high efficiency particulate air (HEPA)-filtered, mass air displacement room in facilities accredited by the American Association for Accreditation of Laboratory Animal Care. Animal rooms were maintained with a light-dark cycle of 12 h (light from 7:00 A.M. to 7:00 P.M.). Rats were acclimated to the exposure conditions by placing them in the Hazleton 1000 chambers and exposing to air for 24 h/day beginning 3 days prior to 2,3-pentanedione exposure. Food was removed for 6 h/day during acclimation.

### Vapor generation and monitoring

2,3-Pentanedione (CAS# 600-14-6) was purchased as a single lot from Acros Organics, Morris Plains, NJ. The purity was ≥ 99.0%. The PD vapor generation and chamber monitoring systems were described previously [3]. The chamber air temperature was maintained at 24 ± 3°C and 40–70% relative humidity throughout the study.

### Inhalation exposures

Rats (n = 5) were exposed to a nominal concentration of 200 ppm PD in Hazleton 1000 exposure chambers, 6 h/d, 5 d/wk for 12 days of exposure. The actual daily PD exposure concentration was 200.12 ± 0.18 ppm (mean ± SD). Control animals (n = 6) were exposed to filtered, conditioned air at the same flow rate in the control chamber. Food was removed during the exposures and water was always available. The animals remained in the chambers for the duration of the study.

### Necropsy, tissue sampling, and laser capture microdissection

Rats were euthanized immediately following the last exposure with Nembutal (100 mg/kg, ip) and exsanguinated via the inferior vena cava. Under RNAse-free conditions, the lungs were removed intact and inflated with an RNAse-free 50/50 mixture of OCT and 0.9% saline. One half of each lobe was frozen at -80°C and the other half was fixed in 10% neutral-buffered formalin for histopathology.

Frozen lung samples were serial sectioned for laser capture microdissection (LCM). The first serial section (6 microns) was stained with hematoxylin and eosin (H&E) and used to locate areas for microdissection (map slide). The next eight serial sections were cut at 8 microns and placed on PET foil slides for LCM. The ninth serial section was cut at 6 microns, stained with H&E and used as a map slide. A total of 45 serial sections were taken in this manner from each lung to assure adequate LCM sampling for microarray analysis.

The Cellcut instrument (Molecular Machines and Industries, Zurich, SW) was used to microdissect the areas of interest. Microdissection was performed within seven days of cutting frozen samples. Samples were kept at -80°C until microdissection. To avoid RNA degradation, only 2 slides were stained at a time to allow for the staining, desiccation, localization of lesions under the microscope, and laser microdissection within the restricted time frame. Since the sections typically contained only 1 or 2 fibrotic lesions, it was necessary to repeat the staining and microdissection process for each lung specimen multiple times over a 1–2 day period in order to harvest sufficient material for the RNA extraction.

**Fig 1:**
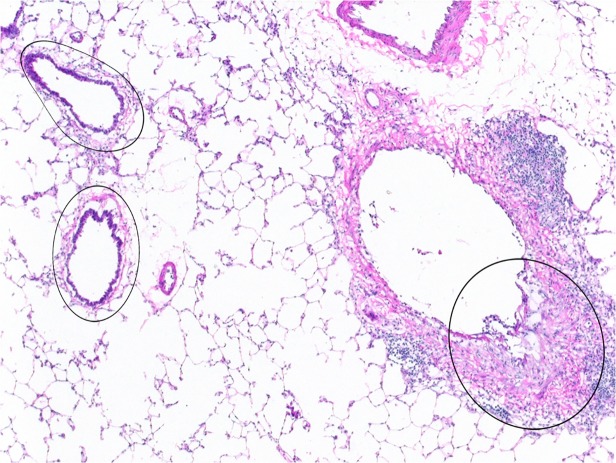
Laser capture microdissection (LCM) of fibrotic and non-fibrotic bronchi. Microdissection of fibrotic bronchial lesions, such as that indicated by the circled area on the right, was performed on frozen sections. Similarly, exposed but non-fibrotic bronchi, such as the two noted on the left, were microdissected and collected separately. RNA was subsequently extracted from both the fibrotic and the non-fibrotic bronchial specimens, and utilized for microarray and PCR analysis. 4x original objective magnification.

Intraluminal fibrotic polyps or fibrotic plaques, along with underlying lamina propria, smooth muscle, and adventitia were microdissected from serial sections of fibrotic bronchi from five PD-exposed rats. Also collected from these same serial sections of 3 exposed rats were non-fibrotic bronchi lined by normal-appearing or reactive regenerative epithelium, with or without mild inflammation, and the underlying lamina propria, muscle, and adventitia (Fig. 1). These non-fibrotic bronchial branches were separate from the fibrotic bronchi to ensure the lack of any fibrosis. Analogous normal bronchial epithelium, underlying lamina propria, muscle, and adventitia were microdissected from serial sections of bronchi from six air-exposed control rats.

### Histopathologic scoring of bronchial fibrosis

The hematoxylin and eosin (H&E) stained frozen section slides from each lung lobe were examined for fibrotic bronchial lesions. Each lesion was categorized as either intraluminal or intramural depending upon the predominant location of the fibrosis. The total number of intraluminal and intramural bronchial fibrotic lesions in all lobes was then added and scored as follows: 1 to 5 lesions = 1+, 6 to 10 = 2+, 11 to 15 = 3+, and >15 = 4+.

### RNA extraction and amplification

Arcturus Picopure RNA extraction buffer (Applied Biosystems, Carlsbad, CA) was used the day of microdissection for lysing each sample. The samples were centrifuged briefly and kept at -80°C until the RNA isolation. RNA isolation was performed within five weeks of microdissection. RNA was extracted from normal tissue and PD-induced lesions. Picoscale RNA was isolated using the PicoPure RNA Isolation Kit (Molecular Devices, Sunnyvale, CA), adapting the protocol for use with CapSure LCM Caps. The amount of isolated RNA (pmole/μl) was determined with a Qubit Instrument. Isolated RNA was analyzed for quality and concentration using a Bioanalyzer PicoChip (Agilent Technologies, Englewood, CO). Twenty (20) ng of total RNA was amplified and labeled using the Nugen WT-Ovation Pico RNA Amplification and Encore Biotin Module protocols.

### Microarray hybridization

Five (5) μg of amplified biotin-aRNAs were fragmented and hybridized to each array for 18 hours at 45°C in a rotating hybridization oven. Array slides were stained with streptavidin/phycoerythrin utilizing a double-antibody staining procedure, washed for antibody amplification according to the GeneChip Hybridization, Wash and Stain Kit user manual, and scanned in an Affymetrix Scanner (Model 3000).

### qPCR validation

Quantitative gene expression levels were detected using real-time PCR with the ABI PRISM 7900HT Sequence Detection System (Applied Biosystems) and TaqMan MGB probes (FAM dye labeled). Primers and probes for all genes analyzed were purchased from Applied Biosystems TaqMan Gene Expression products. For amplification, diluted cDNA was combined with a reaction mixture containing TaqMan universal PCR Master Mix (Applied Biosystems, Catalog No. 4304437) according to manufacturer's instructions. Samples were analyzed in duplicate, and a sample without reverse transcriptase (RT) was included with each plate to detect contamination by genomic DNA. Amplification was carried out as follows: (1) 50°C, 2 min (for uracil-*N*-glycosylase incubation): (2) 95°C, 10 min (denaturation): (3) 95°C, 15 s, 60°C, 30 s (denaturation/amplification) for 40 cycles. Fold increases or decreases in gene expression were determined by quantitation of cDNA from target samples relative to a calibrator sample (control bronchus). The 18S RNA gene was used as the endogenous control for normalization of initial RNA levels. To determine this normalized value, 2^−(ΔΔCt)^ values were compared between target and calibrator samples, where the changes in crossing threshold (ΔCt) = Ct_Target gene_ − Ct_18S RNA_, and ΔΔCt = ΔCt^control^ − ΔCt^target^.

### Immunohistochemistry

Primary Antibodies: Mouse IgG2a anti-smooth muscle actin (SMA) antibody (1:500, Sigma-Aldrich, St Louis, MO), and mouse IgG1 anti-human Tenascin-C antibody (1:500, IBL-America, Inc. Minneapolis, MN). Secondary antibodies: Alexa Fluor 488 Goat anti-mouse IgG1, Alexa Fluor 594 Goat anti-mouse IgG2a, (1:500, Invitrogen, Carlsbad, CA). Permanent sections were cut at 5μ, and blocked with 5% BSA in phosphate-buffered saline (PBS) for non-specific antigen reactivity following citrate buffer retrieval. Primary and secondary antibodies were diluted in blocking solution and incubated overnight at 4°C and room temperature for 90 minutes, respectively. Slides were extensively washed in PBS and cover-slipped with DAPI (Sigma, St Louis, MO) in Fluoromont G mounting media (Southern Biotech, Birmingham, AL). Images were obtained using an Olympus Provis AX70 microscope (Center Valley, PA) equipped with a digital camera and processed using Image-Pro Plus (Media Cybernetics, Silver Spring, MD).

### Data analysis

Raw data were obtained using the Affymetrix GeneChip Command Console Software (AGCC; Version 1.1) using the MAS5 normalization algorithm to generate .CHP files. The resulting data were processed using the Rosetta Resolver system (version7.2) (Rosetta Biosoftware, Kirkland, WA) and the associated Rosetta error model [4]. In order to identify differentially expressed probes, analysis of variance (ANOVA) was used to determine if there was a statistical difference between the means of groups. Specifically, an error-weighted ANOVA and Benjamini-Hochberg multiple test correction with a p value of 0.01 was performed with the Rosetta Resolver system (Version 7.2). A list of differentially expressed genes (DEGs) was generated using a fold change >2 and a p value less than 0.001. The list of differentially expressed genes from the microarray was used as an input for the curated pathway database, Ingenuity Pathway Analysis (IPA; Ingenuity Systems, Redwood City, CA; www.ingenuity.com). IPA’s Core analysis module used the differentially expressed gene set to enrich for canonical and functional pathways or regulatory connections. Significance values were calculated using a right-tailed Fisher’s exact test to determine if a pathway was overrepresented by calculating whether genes in a specific pathway were enriched within the data set compared to all genes on the array in the same pathway at a p<0.05 cutoff for significance based on IPA threshold recommendations. Only pathways with a p value exceeding threshold and having more than two representative genes in the data set were considered. We also identified the enriched biological processes from our differentially expressed gene set by the DAVID database (Database for Annotation, Visualization and Integrated Discovery; http://davidabcc.ncifcrf.gov) with their functional annotational clustering tool using default settings.

The data discussed in this publication have been deposited in NCBI's Gene Expression Omnibus [5] and are accessible through GEO Series accession number GSE52761 (http://www.ncbi.nlm.nih.gov/geo/query/acc.cgi?token=ghexiekyhdclpcf&acc=GSE52761).

## Results

### Histopathology

**Fig 2:**
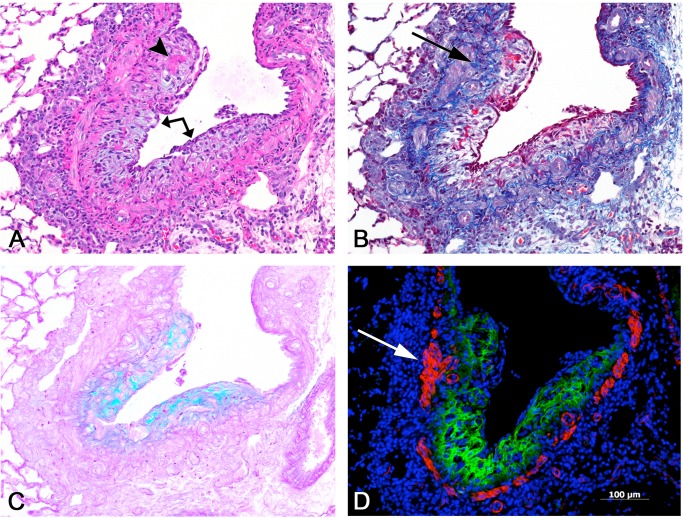
Bronchial fibrosis, intraluminal. Serial sections of a fibrotic bronchus. **A)** A plaque-like proliferation of loose, myxoid, amphophilic connective tissue (arrow) projects into and partially obstructs the lumen of this bronchial branch. The fibrotic lesion is covered by an attenuated, regenerating epithelium, and contains focal deposits of eosinophilic fibrin (arrowhead). A mononuclear cell infiltrate is present in the adventitia. H&E. **B)** The fibrosis is largely devoid of mature collagen, except in the deeper portions of the lesion adjacent to the smooth muscle (arrow). Masson trichrome stain. **C)** The loose, organizing character of the lesion is highlighted by the blue-green web-like reticular pattern demonstrated by the alcian blue stain, indicative of the presence of acidic mucopolysaccharides. **D)** Immunofluorescent stains were used to visualize tenascin C and smooth muscle actin. Extensive deposition of tenascin C (in green) is noted throughout much of the fibrotic lesion. The muscle layer of the bronchial wall is demonstrated in red by the smooth muscle actin antibody; focally, the muscular wall appears to be thickened (arrow). All images, 10X original objective magnification.

All rats exposed to PD developed foci of bronchial fibrosis, primarily within the larger airways, but sometimes extending more distally to the level of the preterminal bronchioles (Table 1). The bronchial fibrotic lesions were principally of the intraluminal plaque-like or polypoid type, although occasional lesions were confined to the bronchial wall (intramural). The stroma of the fibrosis was loose and myxoid, staining with alcian blue for acid mucopolysaccharides and showing little mature collagen with the Masson trichrome stain (Fig. 2). Bronchial and peribronchial inflammatory infiltrates, composed of histiocytes, lymphocytes, eosinophils, and occasional neutrophils were present in the areas of bronchial fibrosis, as well as in some non-fibrotic areas. Bronchial epithelial ulceration, with fibrin deposits on the surface, was often seen in the larger bronchi, and was sometimes extensive. A variety of bronchial epithelial changes were noted in non-ulcerated airways, including reactive and regenerative epithelial changes and epithelial hyperplasia; epithelial hypertrophy and hyperplasia were also seen in some of the bronchioles. Bronchial-associated lymphoid tissue (BALT) was hyperplastic in some animals, particularly in areas of fibrosis. The lungs of air-exposed control rats were unremarkable, except for occasional foci of perivascular or peribronchial eosinophils and mononuclear cells.

**Table 1:** Incidence and Severity of Bronchial Lesions in Rats Exposed to 2,3-Pentanedione.

Bronchial Lesion	0 ppm	200 ppm
Fibrosis	0/6^a^	5/5 (2.0)^b^
Epithelial ulceration	0/6	5/5 (1.6)^c^
Epithelial regeneration	0/6	5/5 (1.8)
Epithelial hyperplasia	0/6	5/5 (1.4)
Inflammation	2/6 (1.0)	5/5 (2.6)

^a^ Incidence of lesion / number of animals examined.

^b^ Fibrosis score (see Materials and Methods).

^c^ Severity score based upon both local severity and the extent of the lesion, using a standard

4 point scale of 1+ = minimal, 2+ = mild, 3+ = moderate, and 4+ = marked.

### Principal Component Analysis

**Fig 3:**
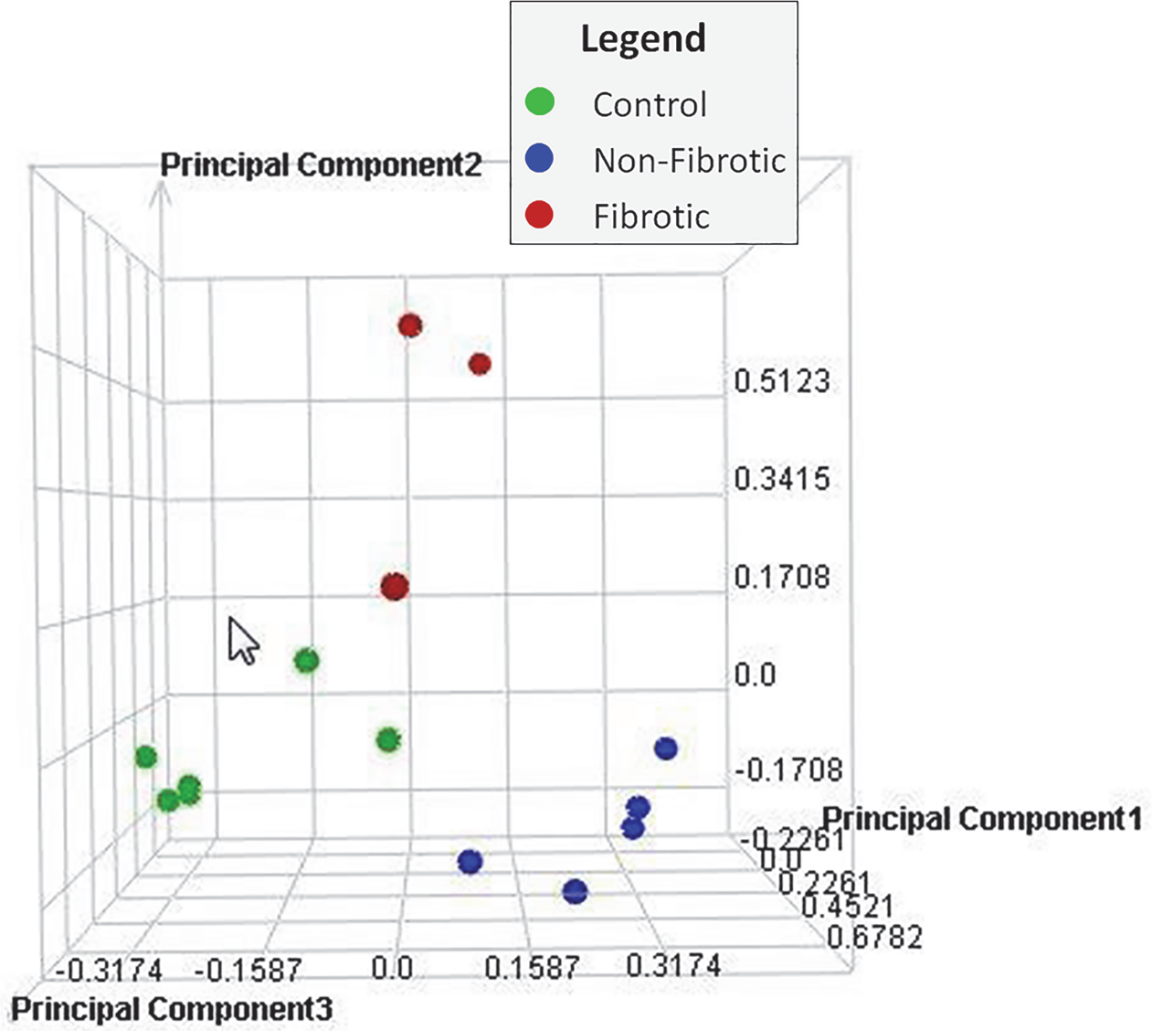
Principal component analysis. Principal Component Analysis (PCA) was performed on all samples and all probes to reduce the dimensionality of the data while preserving the variation in the data set. The PCA indicates that although there is some variability within groups, the samples separate by treatment (air or PD) and sample type (PD-exposed fibrotic; PD-exposed nonfibrotic; air-exposed nonfibrotic).

Principal Component Analysis (PCA) was performed on all samples and all probes to reduce the dimensionality of the data while preserving the variation in the data set (Fig. 3). The PCA indicates that although there is some variability within groups, the samples separate by treatment (air or PD), and sample type (PD-exposed fibrotic; PD-exposed nonfibrotic; air-exposed nonfibrotic).

### Altered Gene Expression in Fibrotic Bronchi of Exposed Rats

**Fig 4:**
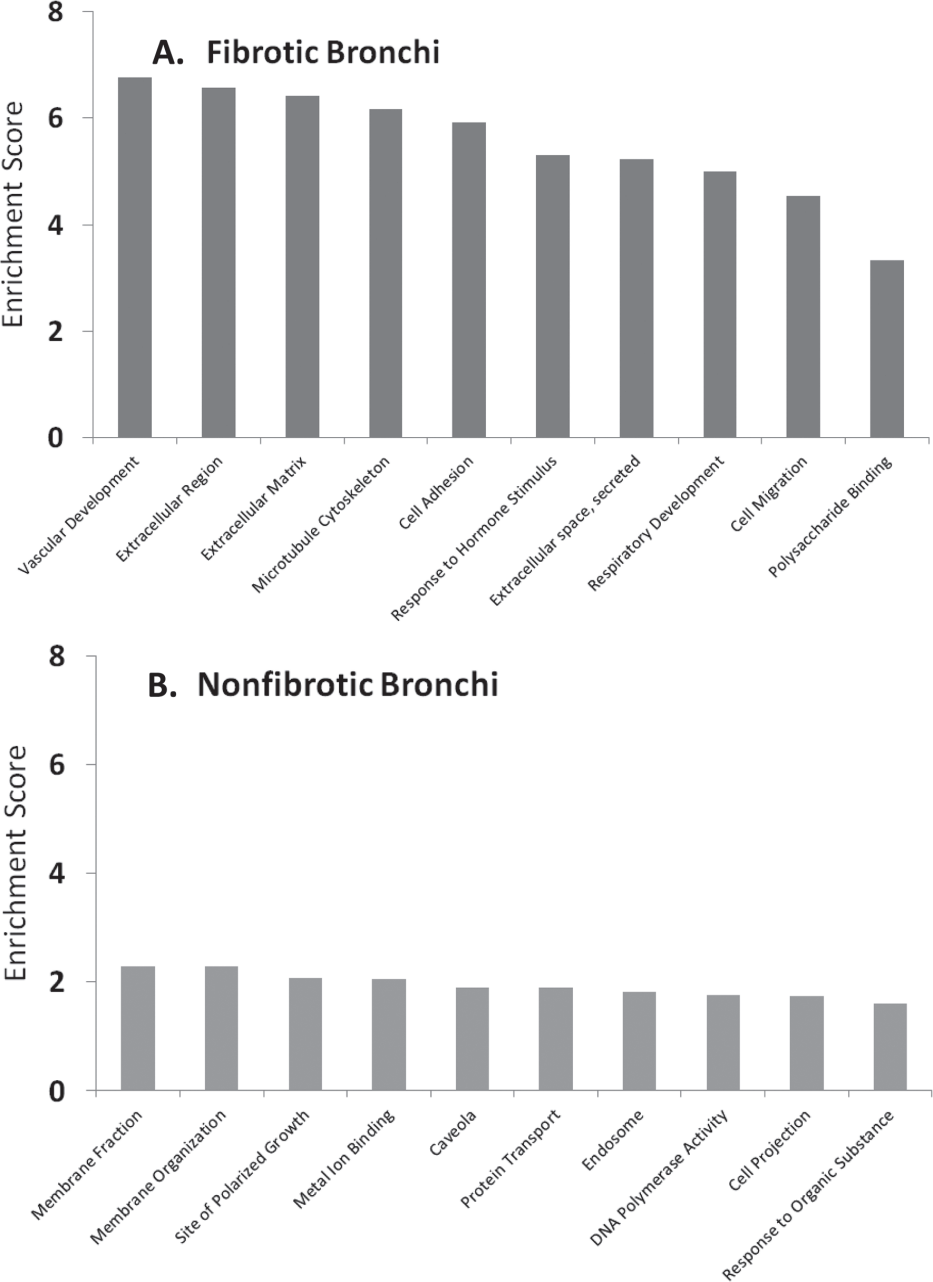
Enriched biological processes. Differentially expressed transcripts from fibrotic bronchi (A) or exposed, non-fibrotic bronchi (B) were enriched for biological processes and cellular functions using the DAVID functional annotation tool. The combined enrichment score is shown on the y-axis for each of the top ten biological categories on the x-axis.

In microdissected samples from fibrotic bronchi, 3807 total genes were significantly (p<0.001) altered relative to bronchial samples from control animals. Functional clustering in the DAVID database enriched for 298 total clusters (S1 Table) of which the top scoring clusters represented vascular development, extracellular matrix, microtubule cytoskeleton, cell adhesion and response to organic substances (Fig. 4A). Genes with the greatest changes in expression (up- or down-regulated) in fibrotic airways are shown in Table 2. Only genes with changes ≥2 fold compared to controls were considered to be biologically significant.

**Table 2:** Fibrotic Bronchi: Major Changes in Gene Expression.

Gene	Gene Description	Fold Increase^a^
Nkain1	Na+/K+ transporting ATPase interacting 1	29.7
Spp1	secreted phosphoprotein 1	29.6
Crabp2	cellular retinoic acid binding protein 2	27.6
Cma1	chymase 1, mast cell	19.2
Lamb3	Laminin, beta 3	18.9
Plau	plasminogen activator, urokinase	18.3
Thbs2	Thrombospondin 2	17.4
Tmem178	Transmembrane protein 178	16.6
Tgm1	transglutaminase 1, K polypeptide	16.4
Bcat1	Branched chain aminotransferase 1, cytosolic	15.7
Plaur	urokinase plasminogen activator receptor	15.4
Serpine1	serpin peptidase inhibitor, clade E, member 1	15.0
Angptl4	Angiopoietin-like 4	14.6
Inhba	inhibin beta-A	14.2
Wisp1	WNT1 inducible signaling pathway protein 1	13.4
		**Fold Decrease** ^**a**^
Sec14l3	SEC14-like 3 (S. cerevisiae)	-90.4
Pon1	Paraoxonase 1	-40.0
Fmo2	Flavin containing monooxygenase 2	-34.9
Ces1d	carboxylesterase 1D	-33.7
Cyp4b1	cytochrome P450, family 4, subfamily b, polypeptide 1	-29.6
Gsta5	Glutathione S-transferase Yc2 subunit	-25.1
Gpr155	G protein-coupled receptor 155	-23.7
Slc15a2	solute carrier family 15 (H+/peptide transporter), member 2	-23.3
Cyp2f4	cytochrome P450, family 2, subfamily f, polypeptide 4	-21.0
Klhl38	Kelch-like 38 (Drosophila)	-20.9
S100a1	S100 calcium binding protein A1	-20.5
Cyp2b1	Cytochrome P450, family 2, subfamily b, polypeptide 1	-20.0
Cdh29	Cadherin-like 29	-20.0
T2	Brachyury 2	-19.1
Hap1	Huntington-associated protein 1	-17.9

^a^Fold change relative to air-exposed controls

Differentially expressed genes (DEGs) for a number of cytokines and growth factors were significantly altered in the fibrotic airways (S2 Table). Several Tgf-β associated genes were significantly up-regulated. The Tgf-β2 isoform was up-regulated approximately 5-fold. A number of interleukins (Il-1α, Il-18, Il-24, Il-33), chemokines (CX3cr1, Cxcr4), and growth factors (Tnfaip6, Ctgf, Egr2, Hbegf, Igfbp4, Ngf, Fn1, Thbs2, and Inhba) were significantly upregulated. A large number of genes coding for extracellular matrix (ECM) components were highly up-regulated in fibrotic airways (S3 Table). Twelve different collagen and procollagen genes were differentially up-regulated from 2- to 13-fold; only Col13a1 was significantly down-regulated (10-fold). In addition, genes for Has2 and Lox, enzymes involved in the synthesis of ECM components, were significantly up-regulated, 13- and 3-fold, respectively.

Gene expression was significantly altered for a number of proteases and protease inhibitors involved in ECM turnover and airway remodeling (S4 Table). Proteases belonging to the Adam, Mmp, and activated kinase gene families were primarily up-regulated in fibrotic bronchi. Plau was the most highly up-regulated protease (18-fold) and its receptor (Plaur) was upregulated 15-fold. Mmp 14 and Mmp2 were upregulated 9- and 2-fold, respectively, and Adam19 was significantly up-regulated 7-fold. A correspondingly large number of serine protease inhibitors (serpins) and tissue inhibitors of Mmps (Timps) were up-regulated in fibrotic lesions. Timp1 and serpine1 were differentially up-regulated 13- and 15-fold, respectively.

DEGs for a number of regulatory matricellular proteins were highly up-regulated in fibrotic airways. Matricellular proteins are non-structural proteins that typically contain binding sites for ECM structural proteins, cell surface receptors, and growth factors. Upregulated matricellular genes included TnN (10-fold), TnC (10-fold), periostin (12-fold), thrombospondin2 (17-fold) and Spp1 (29-fold).

### Altered Gene Expression in Non-fibrotic Bronchi of Exposed Rats

Non-fibrotic tissue devoid of OB lesions was collected from bronchi of three of the five exposed animals. These bronchi were either histologically normal or showed minor epithelial change and/or inflammation, but not ulceration or fibrosis. A total of 4683 genes were differentially expressed (p<0.001) in non-fibrotic bronchi relative to air-exposed controls. A total of only 219 clusters were found in the DAVID database (S5 Table) which showed a different and lower scoring enrichment pattern than fibrotic bronchi. Top scoring pathway enrichments included cell membrane organization, polarized growth, metal and lipoprotein binding, and other generalized responses to chemical exposure (Fig. 4B). The top canonical pathways using IPA pathway analysis in the non-fibrotic airways in PD-exposed animals differed significantly from those predicted for fibrotic bronchi, and included MAPK signaling, STAT3 pathway, acute phase response signaling, and B-cell receptor signaling.

About 2000 DEGs in non-fibrotic bronchi were different from the DEGs in fibrotic bronchi, and 1089 DEGs were common to both fibrotic and nonfibrotic airways (data not shown). Some of the major alterations of genes in non-fibrotic bronchi are shown in Table 3.

**Table 3:** Non-Fibrotic Bronchi: Major Changes in Gene Expression.

Gene	Gene Description	Fold-Increase^a^
Cldn18	Claudin 18	9.4
Gas6	Growth arrest specific 6	9.2
Nog	noggin	8.0
Lrp10	Low-density lipoprotein receptor-related protein 10	5.6
Nrg1	neuregulin 1	5.3
Tubb4	Tubulin, beta 4	4.9
Apln	Apelin	4.7
Slc15a1	solute carrier family 15 (oligopeptide transporter), member 1	4.7
Arhgap17	Rho GTPase activating protein 17	3.9
Clic5	Chloride intracellular channel 5	3.9
Akap5	A kinase (PRKA) anchor protein 5	3.9
Zfp318	Zinc finger protein 318	3.8
Hdac10	Histone deacetylase 10	3.7
Ubr2	Ubiquitin protein ligase E3 component n-recognin 2	3.6
Pdgfrb	Platelet derived growth factor receptor, beta polypeptide	3.5
		**Fold-Decrease** ^**a**^
S1pr1	sphingosine-1-phosphate receptor 1	-5.2
Tlr2	Toll-like receptor 2	-5.3
Rasgrp1	RAS guanyl releasing protein 1 (calcium and DAG-regulated)	-5.4
Stra6	Stimulated by retinoic acid gene 6	-5.5
C9	complement component 9	-5.8
Sbp	spermine binding protein	-6.2
Lgi1	Leucine-rich, glioma inactivated 1	-6.3
Sorl1	Sortilin-related receptor, LDLR class A repeats-containing	-6.4
Spr	sepiapterin reductase	-6.6
Slc23a1	solute carrier family 23 (nucleobase transporters), member 1	-6.9
Zfp853	Zinc finger protein 853	-7.5
Ppara	peroxisome proliferator activated receptor alpha	-7.7
Cyp2a3	cytochrome P450, family 2, subfamily a, polypeptide 3	-8.5
Dmrt2	Doublesex and mab-3 related transcription factor 2	-9.0
Gad1	glutamate decarboxylase 1	-10.1

^a^Fold change relative to air-exposed controls

The differential gene expression for a number of cytokines and growth factors was significantly altered in the non-fibrotic airways (S6 Table). Unlike fibrotic bronchi, most altered cytokines and growth factors were down-regulated in non-fibrotic bronchi. Few genes associated with airway remodeling were up-regulated in the non-fibrotic airways, as expected.

### RT-PCR Confirmation of Microarray Results

**Fig 5:**
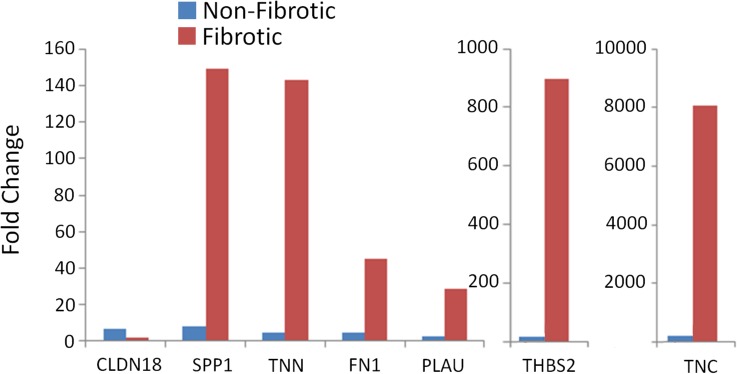
RT-PCR confirmation of microarray data. Confirmation of the microarray results was obtained for a subset of genes by quantitative RT-PCR (qPCR). All changes in gene expression measured by qPCR were in agreement with the changes detected by microarray.

Confirmation of the microarray results was obtained for a subset of genes by quantitative RT-PCR (qPCR). This subset included genes that were up-regulated, and genes with differential expression in fibrotic and non-fibrotic tissue. Up-regulation of gene expression for Spp1, Tnn, Tnc, Fn1, Thbs2, and Plau in fibrotic airways, and up-regulation of Cldn18 in non-fibrotic *airways was also observed by qPCR (Fig. 5).*

**Fig 6:**
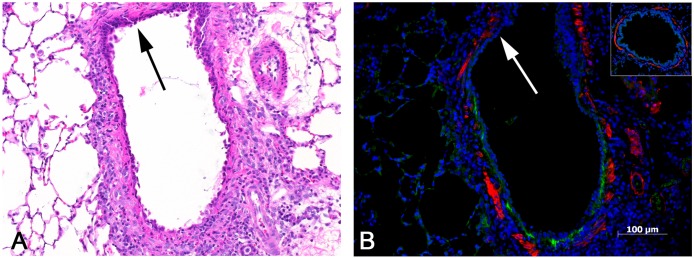
Inflamed, non-fibrotic bronchus with tenascin C expression. **A)** This bronchus shows no evidence of fibrosis, but exhibits a mild infiltrate of mononuclear cells and is lined by an attenuated epithelium with a reactive and regenerative appearance. Absence of inflammation in the upper portion of bronchus (arrow). H&E, 10x original objective magnification. **B)** Early tenascin C expression is indicated by the linear green band located just beneath the epithelial lining. Note that there is no tenascin C expression in the upper portion of the bronchus (arrow), in which there is little or no inflammation and less attenuation of the epithelial lining (compare to A). The inset shows a bronchus from one of the control lungs, with absence of any tenascin C expression. Immunofluorescent stains were used to label tenascin C (green) and smooth muscle actin (red).

The increased expression of Tnc in fibrotic and non-fibrotic airways was also verified by immunohistochemistry (Figs. 2D and 6B). No staining of Tnc was observed in airways from control animals (Fig. 6B, inset). In PD-exposed animals, minimal linear Tnc staining was observed in the subepithelial tissue of the walls of non-fibrotic airways (Fig. 6B), whereas in fibrotic bronchi, areas of intraluminal fibrosis stained heavily for Tnc (Fig. 2D).

## Discussion

In this study we investigated differential gene expression in the airways of animals with PD-induced OB in order to identify potential molecular pathways for this disease. The use of laser capture microdissection to collect only the affected airway tissue provided highly specific and unique information on differentially expressed genes. Over 3800 genes were differentially altered in fibrotic bronchi, demonstrating the complexity of this disease. Cluster analysis indicated significant enrichment for genes involved with vascular development and the remodeling of the ECM in the fibrotic airways. Genes coding for ECM components, proteases and their inhibitors, and matricellular proteins that regulate the ECM were significantly up-regulated in fibrotic airways. These genes appear to be important in the pathogenesis of airway fibrosis and present potential targets for intervention.

Transforming growth factor-β is a key cytokine involved in tissue repair and fibrosis, and increased expression of TGF-βhas been associated with OB in lung transplant patients [6–9]. Tgf-β and related genes were significantly up-regulated in bronchial fibrotic lesions from PD-exposed animals. Pathway analysis indicated potential interactions between the Tgf-βisoforms as well as with many downstream genes that can contribute to OB (S1 Fig.). However, Tgf-β regulates many cellular functions, and the known signaling pathways of this pleiotropic mediator are highly complex. Although the canonical signaling pathway for Tgf-β involves the Smad transcriptional activators, there were no changes in Smad in airways of PD-exposed rats despite the increased expression of Tgf-β.

Although Tgf-β1 generally is considered the predominant isoform involved in development of fibrosis [10], Tgf-β2 was the major isoform that was up-regulated (∼5-fold) in bronchial fibrotic lesions from PD-exposed rats. There is increasing evidence that Tgf-β2 is the primary isoform expressed in the airways [11–14]; however, little is known about the specific functions of Tgf-β2 in airway fibrosis. The results of this study suggest that specific signaling pathways mediated by Tgf-β2 may be important in the development of PD-induced bronchial fibrosis.

A number of interleukins (Il-1α, Il-24, Il-33, Il-18,) were significantly up-regulated in fibrotic airways of PD-exposed rats. Pathway analysis illustrates the extensive interrelationships between these interleukins and other cytokines in the fibrotic airways (S2 Fig.). The Il-1 family of cytokines is well known for its involvement in inflammation and immune regulation. Il-1α (up-regulated ∼8-fold) has been reported to promote fibrosis by stimulating fibroblast proliferation [15] and the synthesis of fibronectin, collagen, Mmps and Timps [16–18]. In addition, Il-1α promotes the synthesis of other pro-fibrotic cytokines including Tgf-β [19].

Il-18 and Il-33, also members of the Il-1 family, were differentially up-regulated 3- and 11-fold, respectively. Both Il-18 and Il-33 have been associated with promotion of systemic Th2 responses [20, 21]. Importantly, Il-18 has also been shown to stimulate fibroblast proliferation and collagen synthesis [22] and has been associated with obstructive lung diseases such as COPD and pulmonary fibrosis [20, 23]. Although Il-33 is constitutively expressed in the epithelium of the bronchus and small airways [24], there is little evidence supporting a role for Il-33 in OB. However, Il-33 is thought to be released by damaged cells undergoing necrosis [25], and like Il-1α, Il-33 may amplify immune responses during tissue injury. These reports suggest that Il-33 may be involved with the initial response to epithelial injury by PD. Interestingly, Il-33 has been reported to reduce antibody-mediated rejection and prolong allograft survival in a cardiac rejection model [26].

Both Tgf-β and Il-1 have been shown to stimulate the production of fibronectins [27, 28]. Fibronectin-1 (Fn1) expression was up-regulated about 13-fold in fibrotic airways of PD-exposed rats relative to controls suggesting a central role in development of OB lesions. Fibronectins are potent fibroblast growth factors [29] secreted by alveolar macrophages, fibroblasts, endothelial cells, alveolar type 2 and bronchial epithelial cells in injured lung tissue. Secreted Fn associates with collagen and other ECM components during tissue repair, and acts as an adhesive for migrating cells with Fn-specific surface receptors [30]. Evaluation of the Fn1 associated pathways (S3 Fig.) illustrates the potential influence Fn1 has on the altered expression of many genes in OB.

Aberrant repair caused by repeated PD exposure appears to involve sustained fibroproliferation and secretion of large quantities of ECM components by myofibroblasts resulting in airway fibrosis. Gene expression for major structural components of the ECM, such as the collagens and laminins, was significantly up-regulated in airways with fibrotic lesions. Hyaluronan synthase 2 (Has2), the enzyme responsible for synthesizing the ECM component hyaluronan, was significantly elevated 13-fold, suggesting the potential importance of this ECM protein in OB. Lysyl oxidase (Lox) is the primary collagen-crosslinking enzyme and has been associated with the irreversible phase of fibrosis. Expression of Lox and related genes was increased 2 to 5-fold in fibrotic lesions from PD-exposed rats, and was reported to be significantly elevated in biopsy samples from OB patients [31].

The proteases and their inhibitors are major counterbalancing regulators of ECM turnover. Alterations in the balance of proteases and their inhibitors have been reported in OB patients [32, 33] and in animal models of OB [34, 35]. A number of proteases including the matrix metalloproteinases (Mmp2, Mmp14), plasminogen activator urokinase (Plau) and its receptor (Plaur), a disintegrin and metalloproteinase 19 (Adam19), and serine protease22 (Prss22) were highly up-regulated in PD-exposed fibrotic airways, possibly in response to the increased synthesis of ECM components. The proteases have many functions in addition to degrading ECM, and can activate cytokines, growth factors, and their receptors by enzymatic cleavage of latent forms. Proteases including Mmp2 and Mmp9 are known to cleave latent Tgf-β to an active form [36, 37]. Elevated Plaur expression has been reported for tissues undergoing extensive remodeling [38] and is thought to localize activation of plasminogen and generation of plasmin at the cell surface for degradation of ECM.

The serine peptidase inhibitors (serpins) are the major source of protease inhibition in the lung [39], and are important in maintaining a balance between essential protease activity and protecting against protease mediated damage. Gene expression for a number of serpins was significantly up-regulated in the fibrotic lesions. An additional level of control over excess protease activity is provided by tissue inhibitors of Mmps (Timps) which can block proteolytic activity. Timp-1 was up-regulated ∼13-fold in fibrotic lesions.

Matricellular proteins are nonstructural ECM proteins that regulate cell function by influencing cell adhesion, migration, proliferation, and apoptosis. Dysregulation of matricellular proteins may contribute to aberrant tissue repair and fibrosis [40, 41]. Genes coding for the matricellular proteins tenascin C (Tnc), tenascin N (TnN), thrombospondin-2 (Thbs2), Spp1 (osteopontin) and periostin (Postn) were among the most highly up-regulated genes in fibrotic airways (11- to 30-fold). Gene expression for the tenascins, matricellular proteins associated with the subepithelial basement membrane, was up-regulated 11-fold in fibrotic airways of PD-exposed rats. Tenascin N is generally associated with the ECM of neurons and its presence in the lung has not previously been reported. Little or no expression of TNC is found in normal adult lung; however, prominent Tnc expression has been reported in animal models of acute lung injury [42, 43], in human idiopathic pulmonary fibrosis [44], COPD [45], and asthma [46]. Pathway analysis of Tnc and other differentially expressed genes in fibrotic airways indicates a Tnc interaction with Fn1, as well as with several proteases (S4 Fig.). In addition to its potential role in tissue repair, TNC may contribute to inhibition of T-cell activation [47] suggesting a potential protective mechanism against transplant rejection during OB development [48]. Recent studies suggest that Tnc modulates the effect of Tgf-β on fibroblast differentiation and collagen synthesis [49]. Increased immunostaining for Tnc was reported previously for diacetyl-treated rats with OB-like lesions [50]. Importantly, increased TNC expression has been observed in airway epithelium of lung transplant patients, with the greatest expression occurring during the early development of OB [48].

Another matricellular protein, Thbs2, was highly up-regulated (∼17-fold) in the bronchial fibrotic lesions. Although the functions of Thbs2 are not completely understood, it is best known for its anti-angiogenic activity [51, 52] and its ability to modulate ECM interactions [53]. Of particular relevance to OB, Thbs2 is thought to interact with ECM components and support calcium-dependent cell attachment [54]. *In vitro* studies indicate that Thbs2 may down-regulate the expression of Mmp-2 and Mmp-9 [55, 56]. Spp1 (osteopontin), another matricellular protein and component of the ECM, also was highly up-regulated (30-fold) in fibrotic bronchi. Spp1 is an adhesive ECM protein that also plays a significant role in integrin-mediated cell signaling. Pathologically elevated levels of Spp1 reportedly promote chronic inflammation and Tgf-β-mediated fibrosis [40, 41].

Not all airways are fibrotic in transplant-related OB, or in animals with OB induced by PD exposure. The non-fibrotic bronchi from PD-exposed rats had normal or mildly altered epithelium, yet differential gene expression was significantly altered for approximately 4683 genes in these airways. Few known pro-fibrotic genes were altered in non-fibrotic bronchi, although several genes associated with acute lung injury were significantly up-regulated. Claudin 18 (Cld18; up-regulated 9-fold), a gene associated with the barrier function of epithelial tight junctions in the lung has been reported to be increased during acute lung injury [57–59]. Similarly, growth arrest-specific-6 (Gas6; up-regulated 9-fold) has been shown to be involved in early injury pathways such as inflammation, cell proliferation, apoptosis, leukocyte migration, and platelet aggregation [60, 61]. Interestingly, the secreted polypeptide noggin, (up-regulated 8-fold), has been reported to bind and inactivate bone morphogenic proteins (BMPs) suggesting an inhibitory role in airway fibrosis. BMPs are members of the Tgf-β superfamily and binding with noggin inhibits them from binding and activating BMP receptors, thus blocking Smad-dependent and non-Smad signaling [62]. Lrp-10 was up-regulated ∼6-fold in non-fibrotic bronchi from exposed rats. The functions of Lrp-10 are not clear, although it has been reported to be a negative regulator of the Wnt/β-catenin pathway [63]. Because Wnt signaling potently stimulates fibroblast activation and production of ECM [64], it is speculated that up-regulation of Lrp10 may be a cellular response to reduce fibroblast activity.

In summary, this report confirms the involvement of several known pro-fibrotic mediators and identifies several highly up-regulated genes not previously associated with OB. Tgf-β2, Il1α and fibronectin are known pro-fibrotic mediators that appear to have key roles in PD-induced bronchial fibrosis. In addition, other genes with little previous association with OB were highly up-regulated in fibrotic airways. Although the magnitude of gene expression does not necessarily correlate with downstream effects, further investigation of these highly up-regulated genes may provide important information needed to unravel the complex mechanism(s) involved in OB. These data are being further evaluated to identify key genes and pathways in the pathogenesis of OB and to facilitate the development of treatment strategies.

## Supporting Information

S1 Fig TGF-β and downstream genes in fibrotic bronchi. TGF-β regulated pathways altered in fibrotic lesions after PD exposure. Microarray analysis was performed on laser capture microdissected bronchial fibrotic lesions from PD exposed rats and was compared to bronchial tissues from air controls. Differentially expressed genes (DEGs) in fibrotic lesions were analyzed by the IPA’s Core analysis. TGF-β genes (bold text) were selected for pathway analysis using the ‘Grow’ tool to display annotated regulatory relationships and interactions. Starting with induction of each TGF-β gene in the center, DEGs from microarray analysis were used to grow and interconnect downstream-dependent genes (red, up-regulated; green, down-regulated).
(TIF)

S2 Fig Interleukin and chemokine pathways in fibrotic bronchi. Interleukin and chemokine regulated pathways altered in fibrotic bronchi after PD exposure. Microarray analysis was performed on laser capture microdissected fibrotic bronchi from PD exposed rats and was compared to bronchial tissues from air controls. Differentially expressed genes (DEGs) in fibrotic bronchi were analyzed by the IPA’s Core analysis. Interleukin (IL1α, IL1RN, IL11, IL18, IL24 and IL33) genes and a chemokine (CCL13) shown in bold text were selected for pathway analysis using the ‘Grow’ tool to display annotated regulatory relationships and interactions. Starting with induction of each selected gene in the center, DEGs from microarray analysis were used to grow and interconnect downstream-dependent genes (red, up-regulated; green, down-regulated).
(TIF)

S3 Fig Fibronectin-1 associated pathways in fibrotic bronchi. Fibronectin regulated pathways altered in fibrotic bronchi after PD exposure. Microarray analysis was performed on laser capture microdissected fibrotic bronchi from PD exposed rats and was compared to bronchial tissues from air controls. Differentially expressed genes (DEGs) in fibrotic bronchi were analyzed by the IPA’s Core analysis. Fibronectin (FN1) as shown in bold text was selected for pathway analysis using the ‘Grow’ tool to display annotated regulatory relationships and interactions. Genes in red were up-regulated and in green were down-regulated in fibrotic bronchi. Genes are displayed in their principal subcellular location in the extracellular space, cell membrane, cytoplasm or nucleus.
(TIF)

S4 Fig Tenascin C associated pathways in fibrotic bronchi. Tenascin C regulated pathways altered in fibrotic bronchi after PD exposure. Microarray analysis was performed on laser capture microdissected fibrotic bronchi from PD exposed rats and was compared to bronchial tissues from air controls. Differentially expressed genes (DEGs) in fibrotic lesions were analyzed by the IPA’s Core analysis. Tenascin C (TNC) as shown in bold text was selected for pathway analysis using the ‘Grow’ tool to display annotated regulatory relationships and interactions. Genes in red were up-regulated and in green were down-regulated in fibrotic bronchi.
(TIF)

S1 Table DAVID Functional Annotation Clustering for DEG found in Fibrotic Bronchi. (XLSX)

S2 Table Fibrotic Bronchi: Differential Expression of Cytokine and Growth Factor Genes. (DOCX)

S3 Table Fibrotic Bronchi: Differential Expression of Extracellular Matrix Genes. (DOCX)

S4 Table Fibrotic Bronchi: Differential Expression of Protease and Protease Inhibitor Genes. (DOCX)

S5 Table DAVID Functional Annotation Clustering for DEGs Found in Non-fibrotic Bronchi. (XLSX)

S6 Table Exposed, Non-fibrotic Bronchi: Differential Expression of Cytokine and Growth Factor Genes. (DOCX)
