# Supplementary figures and images for: Gene Expression in Obliterative Bronchiolitis-Like Lesions in 2,3-Pentanedione-Exposed Rats
Source: PLoS One. 2015 Feb 24;10(2):e0118459. doi: 10.1371/journal.pone.0118459 (PMC4339611; doi:10.1371/journal.pone.0118459)

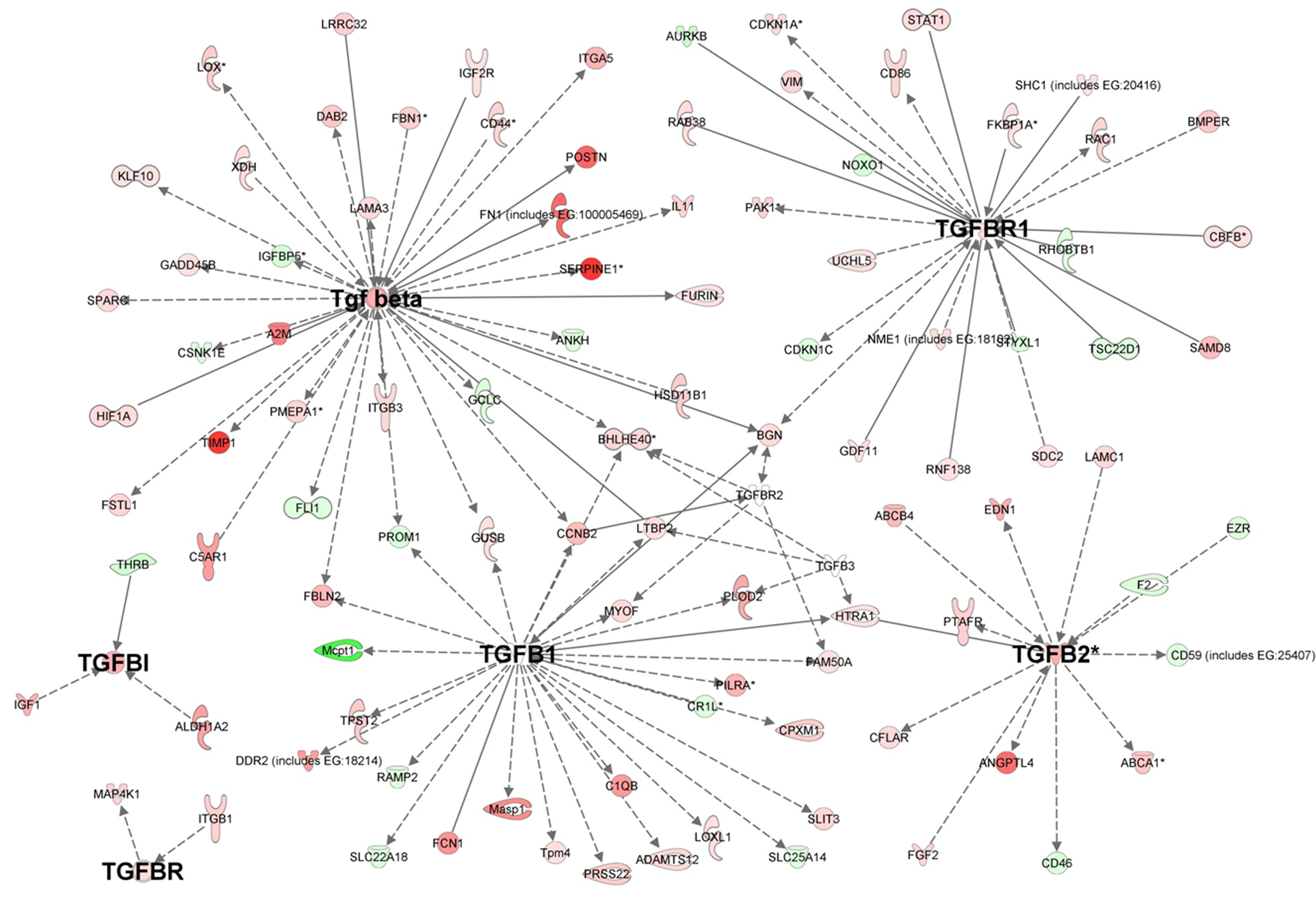

Supplement: S1 Fig — TGF-β regulated pathways altered in fibrotic lesions after PD exposure. Microarray analysis was performed on laser capture microdissected bronchial fibrotic lesions from PD exposed rats and was compared to bronchial tissues from air controls. Differentially expressed genes (DEGs) in fibrotic lesions were analyzed by the IPA’s Core analysis. TGF-β genes (bold text) were selected for pathway analysis using the ‘Grow’ tool to display annotated regulatory relationships and interactions. Starting with induction of each TGF-β gene in the center, DEGs from microarray analysis were used to grow and interconnect downstream-dependent genes (red, up-regulated; green, down-regulated). (TIF) [file pone.0118459.s001.tif]

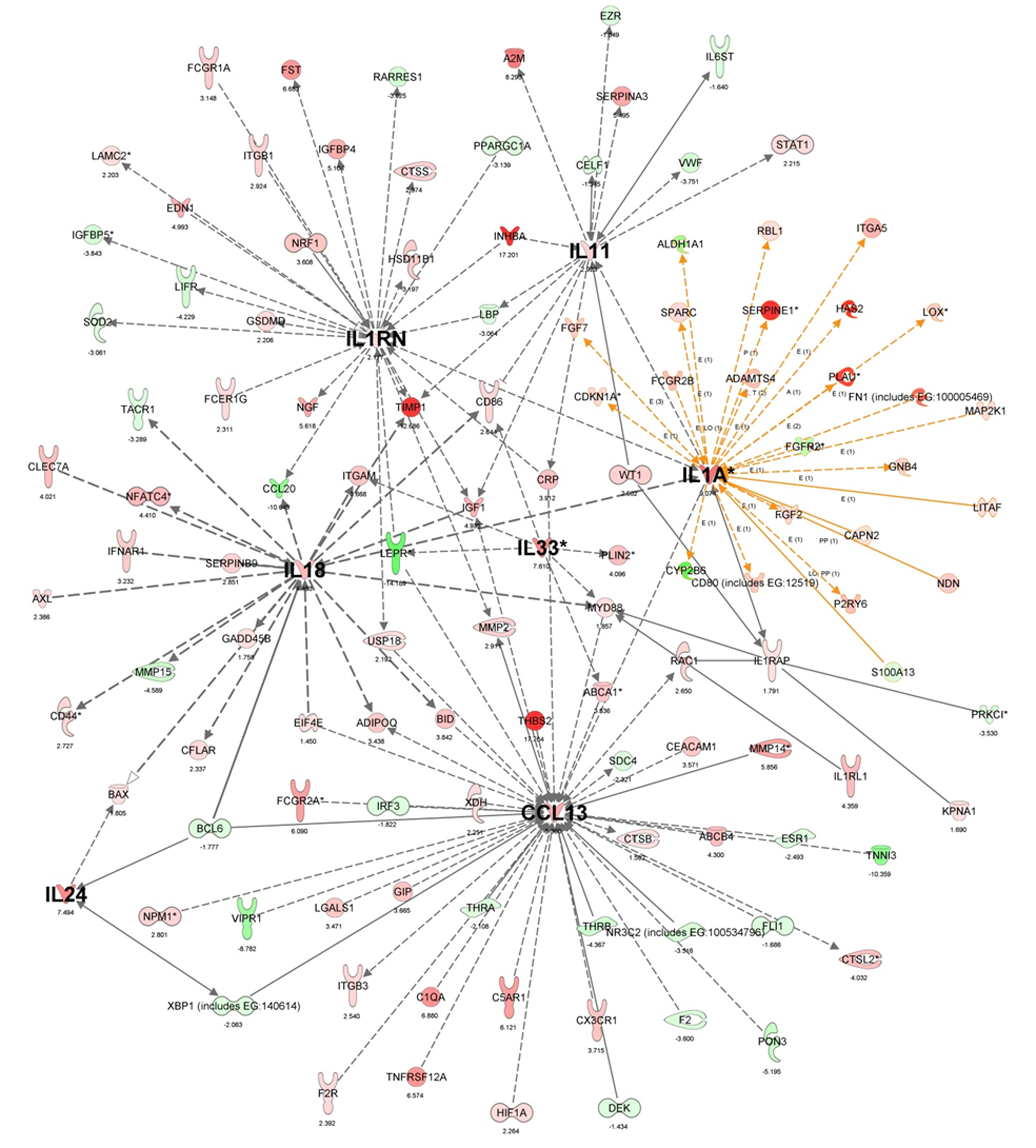

Supplement: S2 Fig — Interleukin and chemokine regulated pathways altered in fibrotic bronchi after PD exposure. Microarray analysis was performed on laser capture microdissected fibrotic bronchi from PD exposed rats and was compared to bronchial tissues from air controls. Differentially expressed genes (DEGs) in fibrotic bronchi were analyzed by the IPA’s Core analysis. Interleukin (IL1α, IL1RN, IL11, IL18, IL24 and IL33) genes and a chemokine (CCL13) shown in bold text were selected for pathway analysis using the ‘Grow’ tool to display annotated regulatory relationships and interactions. Starting with induction of each selected gene in the center, DEGs from microarray analysis were used to grow and interconnect downstream-dependent genes (red, up-regulated; green, down-regulated). (TIF) [file pone.0118459.s002.tif]

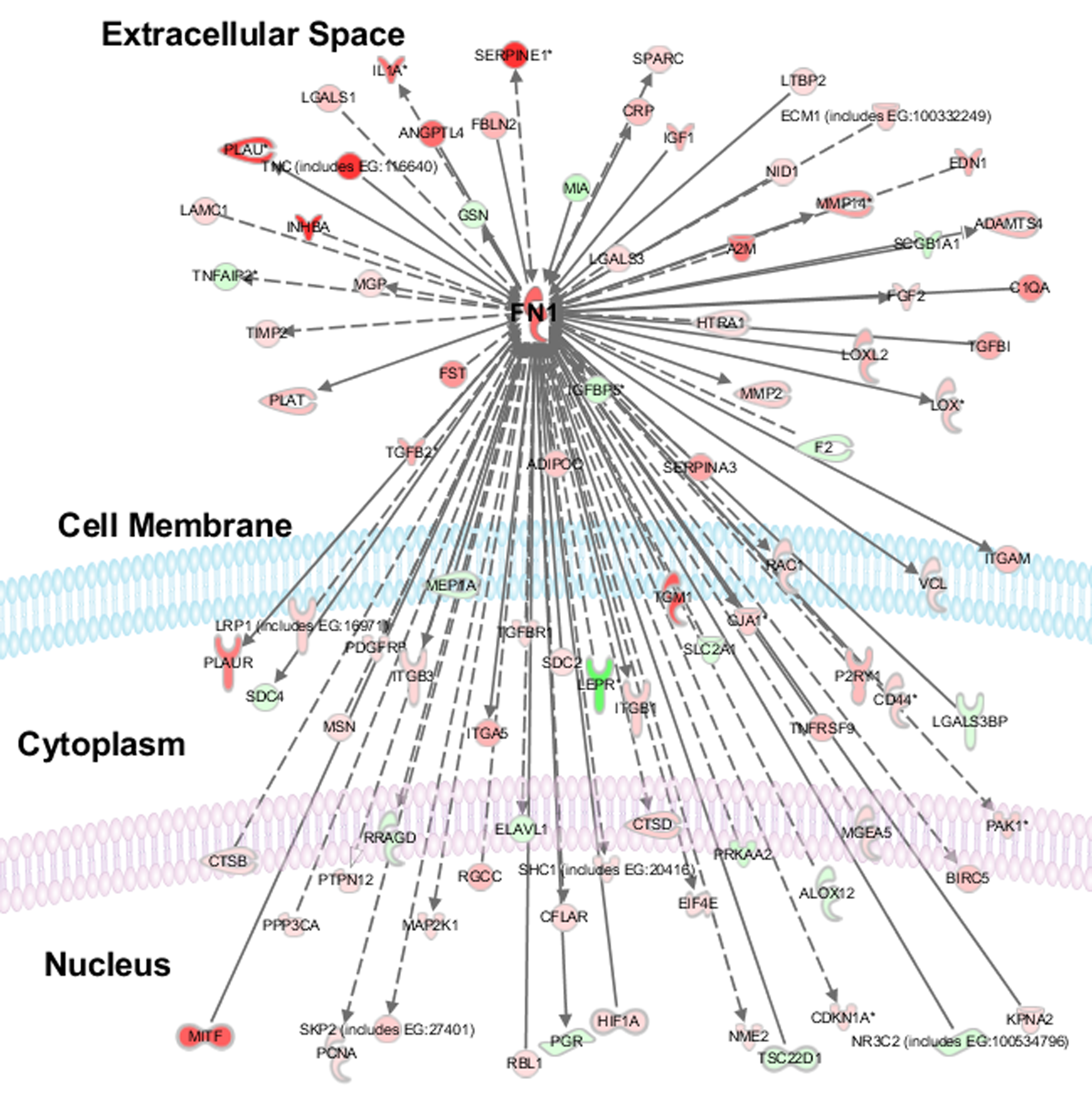

Supplement: S3 Fig — Fibronectin regulated pathways altered in fibrotic bronchi after PD exposure. Microarray analysis was performed on laser capture microdissected fibrotic bronchi from PD exposed rats and was compared to bronchial tissues from air controls. Differentially expressed genes (DEGs) in fibrotic bronchi were analyzed by the IPA’s Core analysis. Fibronectin (FN1) as shown in bold text was selected for pathway analysis using the ‘Grow’ tool to display annotated regulatory relationships and interactions. Genes in red were up-regulated and in green were down-regulated in fibrotic bronchi. Genes are displayed in their principal subcellular location in the extracellular space, cell membrane, cytoplasm or nucleus. (TIF) [file pone.0118459.s003.tif]

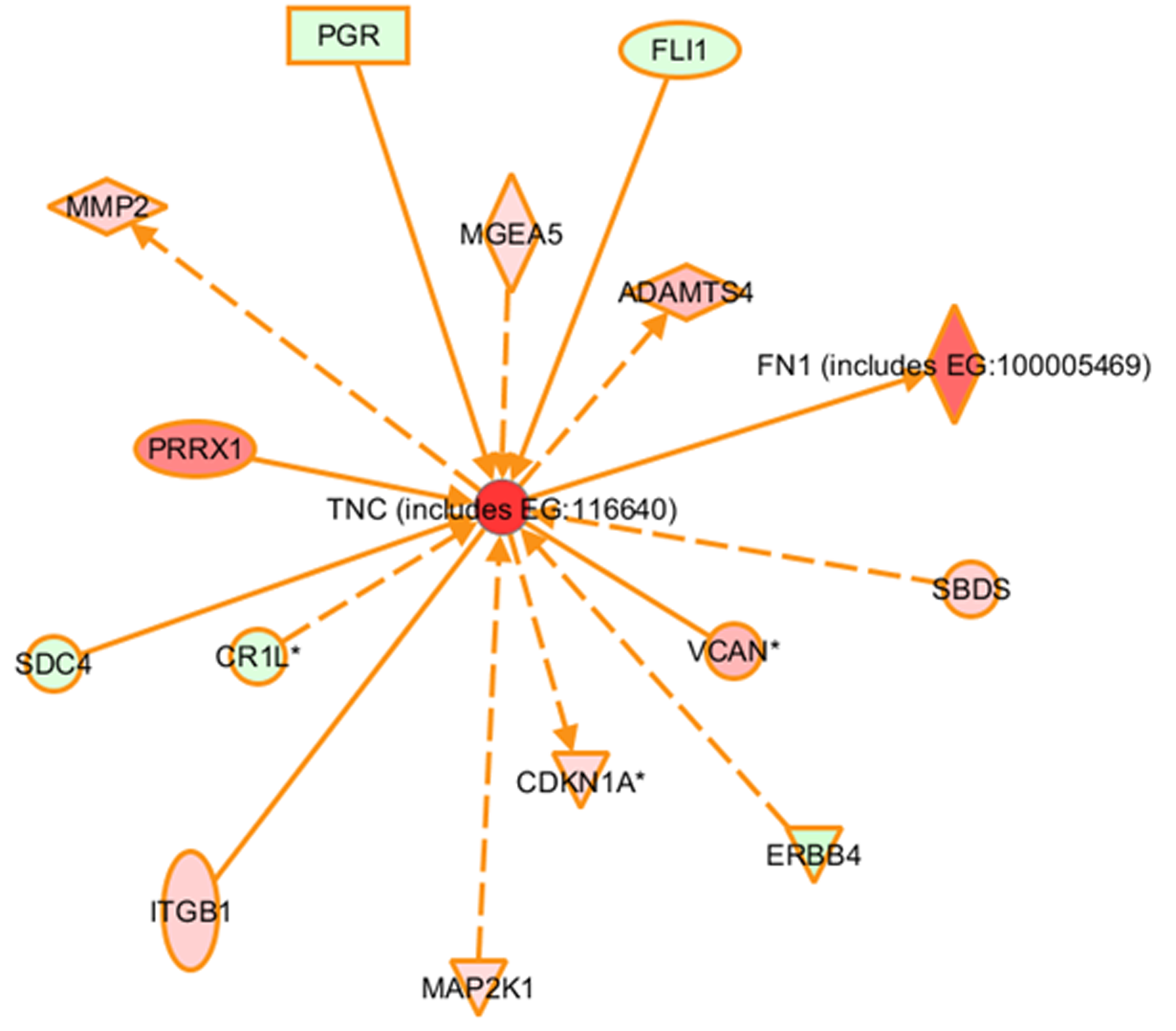

Supplement: S4 Fig — Tenascin C regulated pathways altered in fibrotic bronchi after PD exposure. Microarray analysis was performed on laser capture microdissected fibrotic bronchi from PD exposed rats and was compared to bronchial tissues from air controls. Differentially expressed genes (DEGs) in fibrotic lesions were analyzed by the IPA’s Core analysis. Tenascin C (TNC) as shown in bold text was selected for pathway analysis using the ‘Grow’ tool to display annotated regulatory relationships and interactions. Genes in red were up-regulated and in green were down-regulated in fibrotic bronchi. (TIF) [file pone.0118459.s004.tif]
