# Supplementary material for: Gene Expression in Obliterative Bronchiolitis-Like Lesions in 2,3-Pentanedione-Exposed Rats
Source: PLoS One. 2015 Feb 24;10(2):e0118459. doi: 10.1371/journal.pone.0118459 (PMC4339611; doi:10.1371/journal.pone.0118459)
Supplement: S2 Table — (DOCX) [file pone.0118459.s006.docx]

**Table S2. Fibrotic Bronchi: Differential Expression of Cytokine and Growth Factor Genes**

| **Gene** | **Gene Description** | **Fold Change^a^** |
| --- | --- | --- |
| Cx3cr1 | chemokine (C-X3-C motif) receptor 1 | 4.2 |
| Cxcl3 | chemokine (C-X-C motif) ligand 3 | -7.1 |
| Cxcr4 | Chemokine (C-X-C motif) receptor 4 | 3.0 |
| Il1a | interleukin 1 alpha | 8.4 |
| Irak1bp1 | Interleukin-1 receptor-assoc kinase 1 binding protein 1 | -2.9 |
| Il1rn | interleukin 1 receptor antagonist | 2.6 |
| Il18 | interleukin 18 | 3.2 |
| Il24 | interleukin 24 | 11.9 |
| Il33 | Interleukin 33 | 10.6 |
| Tnfrsf12a | Tumor necrosis factor receptor superfamily, member 12a | 7.0 |
| Tnfaip6 | tumor necrosis factor alpha induced protein 6 | 9.2 |
| Tgfbi | Transforming growth factor, beta induced | 3.5 |
| Tgfb2 | transforming growth factor, beta 2 | 4.6 |
| Thbs2 | Thrombospondin 2 | 17.4 |
| Inhba | inhibin beta-A | 14.2 |
| Bmp3 | bone morphogenetic protein 3 | -3.1 |
| Ctgf | connective tissue growth factor | 5.7 |
| Egfr | epidermal growth factor receptor | 2.5 |
| Egr2 | early growth response 2 | 7.2 |
| Fgfr2 | fibroblast growth factor receptor 2 | -6.9 |
| Fgfr3 | Fibroblast growth factor receptor 3 | -3.0 |
| Fn1 | Fibronectin 1 | 12.9 |
| Grb10 | Growth factor receptor bound protein 10 | 2.8 |
| Hbegf | heparin-binding EGF-like growth factor | 6.3 |
| Igf1 | insulin-like growth factor 1 | 3.7 |
| Igfbp4 | Insulin-like growth factor binding protein 4 | 4.8 |
| Igfbp5 | Insulin-like growth factor binding protein 5 | -4.6 |
| Ngf | Nerve growth factor (beta polypeptide) | 7.4 |

**^a^**Fold change relative to air-exposed controls
