# Supplementary material for: Gene Expression in Obliterative Bronchiolitis-Like Lesions in 2,3-Pentanedione-Exposed Rats
Source: PLoS One. 2015 Feb 24;10(2):e0118459. doi: 10.1371/journal.pone.0118459 (PMC4339611; doi:10.1371/journal.pone.0118459)
Supplement: S3 Table — (DOCX) [file pone.0118459.s007.docx]

**Table S3. Fibrotic Bronchi: Differential Expression of Extracellular Matrix Genes**

| **Gene** | **Sequence Description** | **Fold-change** ^a^ |
| --- | --- | --- |
| Col1a2 | Collagen, type I, alpha 2 | 2.2 |
| Col4a1 | Collagen, type IV, alpha 1 | 2.7 |
| Col5a1 | Collagen, type V, alpha 1 | 5.0 |
| Col5a2 | Collagen, type V, alpha 2 | 4.1 |
| Col5a3 | collagen, type V, alpha 3 | 4.8 |
| Col6a2 | Collagen, type VI, alpha 2 | 2.7 |
| Col12a1 | collagen, type XII, alpha 1 | 13.4 |
| Col13a1 | Collagen, type XIII, alpha 1 | -9.7 |
| Col14a1 | Collagen, type XIV, alpha 1 | 3.0 |
| Col18a1 | Collagen, type XVIII, alpha 1 | 2.8 |
| Cthrc1 | Collagen triple helix repeat containing 1 | 8.9 |
| Plod2 | Procollagen lysine, 2-oxoglutarate 5-dioxygenase 2 | 6.1 |
| Fbn1 | Fibrillin 1 | 3.0 |
| Has2 | hyaluronan synthase 2 | 12.7 |
| Itgb1 | integrin, beta 1 | 3.0 |
| Itgb3 | Integrin, beta 3 | 4.2 |
| Itga5 | Integrin, alpha 5 (fibronectin receptor, a polypeptide) | 5.5 |
| Itga6 | Integrin, alpha 6 | 4.5 |
| Lamb3 | Laminin, beta 3 | 18.9 |
| Lamc2 | Laminin, gamma 2 | 3.1 |
| Lamc3 | Laminin gamma 3 | -6.4 |
| Lox | Lysyl oxidase | 4.5 |
| Loxl2 | Lysyl oxidase-like 2 | 4.2 |
| Spon1 | spondin 1, extracellular matrix protein | 5.2 |
| Tnc | Tenascin C | 11.0 |
| Tnn | Tenascin N | 10.8 |

^a^ Fold change relative to air-exposed controls
