# Supplementary material for: Gene Expression in Obliterative Bronchiolitis-Like Lesions in 2,3-Pentanedione-Exposed Rats
Source: PLoS One. 2015 Feb 24;10(2):e0118459. doi: 10.1371/journal.pone.0118459 (PMC4339611; doi:10.1371/journal.pone.0118459)
Supplement: S4 Table — (DOCX) [file pone.0118459.s008.docx]

**Table S4. Fibrotic Bronchi: Differential Expression of Protease and Protease**

**Inhibitor Genes**

| **Gene** | **Sequence Description** | **Fold-Change^a^** |
| --- | --- | --- |
| Adam8 | ADAM metallopeptidase domain 8 | -9.0 |
| Adam10 | ADAM metallopeptidase domain 10 | 2.0 |
| Adam19 | A disintegrin and metallopeptidase domain 19 (meltrin beta) | 6.8 |
| Mmp2 | matrix metallopeptidase 2 | 2.3 |
| Mmp14 | matrix metallopeptidase 14 (membrane-inserted) | 3.1 |
| Mmp24 | Matrix metallopeptidase 24 | -2.1 |
| Timp1 | TIMP metallopeptidase inhibitor 1 | 13.3 |
| Timp2 | TIMP metallopeptidase inhibitor 2 | 2.1 |
| Timp4 | Tissue inhibitor of metalloproteinase 4 | -4.8 |
| Plat | plasminogen activator, tissue | 3.9 |
| Plau | plasminogen activator, urokinase | 18.3 |
| Plau | plasminogen activator, urokinase | 9.6 |
| Plaur | urokinase plasminogen activator receptor | 15.4 |
| Prss22 | Protease, serine, 22 | 5.4 |
| Serpine1 | serpin peptidase inhibitor, clade E member 1 | 15.0 |
| Serpinf1 | Serpin peptidase inhibitor, clade F member 1 | 2.3 |
| Serping1 | Serine (or cysteine) peptidase inhibitor, clade G, member 1 | 2.1 |
| Serpinh1 | Serine (or cysteine) peptidase inhibitor, clade H, member 1 | 2.5 |
| Serpinb5 | serpin peptidase inhibitor, clade B (ovalbumin), member 5 | -2.8 |

^a^ Fold change relative to air-exposed controls
