# Supplementary material for: Gene Expression in Obliterative Bronchiolitis-Like Lesions in 2,3-Pentanedione-Exposed Rats
Source: PLoS One. 2015 Feb 24;10(2):e0118459. doi: 10.1371/journal.pone.0118459 (PMC4339611; doi:10.1371/journal.pone.0118459)
Supplement: S6 Table — (DOCX) [file pone.0118459.s010.docx]

**Table S6. Exposed, Non-fibrotic Bronchi: Differential Expression of Cytokine**

**and Growth Factor Genes**

| **Gene** | **Description** | **Fold Change^a^** | **Family** |
| --- | --- | --- | --- |
| Gas6 | Growth arrest specific 6 | 9.2 | growth factor |
| Igf2bp2 | Insulin-like growth factor 2 mRNA binding protein 2 | 2.8 | growth factor |
| Nrg1 | neuregulin 1 | 5.3 | growth factor |
| Ccl9 | Chemokine (C-C motif) ligand 9 | -3.4 | cytokine |
| Cxcl1 | chemokine (C-X-C motif) ligand 1 | -2.2 | cytokine |
| Cxcl3 | chemokine (C-X-C motif) ligand 3 | -2.4 | cytokine |
| Cxcr4 | chemokine (C-X-C motif) receptor 4 | -4.4 | cytokine |
| Fndc3b | Fibronectin type III domain containing 3B | -3.4 | growth factor |
| Fbln1 | Fibulin 1 | -2.2 | growth factor |
| Ghitm | Growth hormone inducible transmembrane protein | -2.2 | growth factor |
| Igfbp5 | Insulin-like growth factor binding protein 5 | -3.1 | growth factor |
| Thbs4 | thrombospondin 4 | -2.4 | cytokine |
| Cd36 | CD36 molecule (thrombospondin receptor) | 2.3 | cytokine |

**^a^**Fold change relative to air-exposed controls
